# Assessment of Asphaltene and Maltene Interfacial Interactions with Antifoam Additives: A Rheological Approach

**DOI:** 10.1021/acsomega.5c00847

**Published:** 2025-05-14

**Authors:** Mariana T. Mendes, Andressa O. dos Santos, Rafael F. Perez, Osvaldo Karnitz Junior, Claudia R. E. Mansur

**Affiliations:** 1 Programa de Engenharia Metalúrgica e de Materiais-PEMM/COPPE, 28125 Universidade Federal do Rio de Janeiro, Av. Horácio Macedo, 2030 - Bloco F-CT, Cidade Universitária, Rio de Janeiro − RJ 21941-598, Brazil; 2 Instituto de Macromoléculas/Laboratório de Macromoléculas e Colóides na Indústria de Petróleo, 28125 Universidade Federal do Rio de Janeiro, Rua Moniz Aragão, 360. Bloco 8G-CT2, Cidade Universitária, Rio de Janeiro − RJ 21941-594, Brazil; 3 Leopoldo Américo Miguez de Mello Research and Development Center, Rio de Janeiro − RJ BR 21941-598, Brazil

## Abstract

Polydimethylsiloxane (PDMS)-based oil antifoams are extensively employed, but still, their impact on oil remains inconclusive due to the intricate nature and diverse composition of the oil. So far, the literature declares asphaltenes as the main contributors to foam stabilization followed by resins, along with short-chain carboxylic acids, solids, and even naphthenic acids. The present work aims to study the influence of antifoam formulations containing silicones of different molar masses on the oil/air interface, as well as evaluate its influence on the fractions separately, that is, on asphaltenes, on the model solution/air interface, and also on resins, examining the maltene/air interface. Hence, the interfacial rheology technique utilizing the Double Wall Ring (DWR) accessory was employed to assess the viscoelastic behavior of oil and its constituents (asphaltenes and maltenes) in the presence of antifoam products. This approach aims to gain insight into how these products interact with the oil–air interface and the various fractions of the oil. The findings from the DWR accessory align with the results of the foam formation tests, indicating that the products demonstrating superior efficacy in reducing the initial foam height also played a role in decreasing the interfacial elastic modulus of the respective systems.

## Graphical abstract

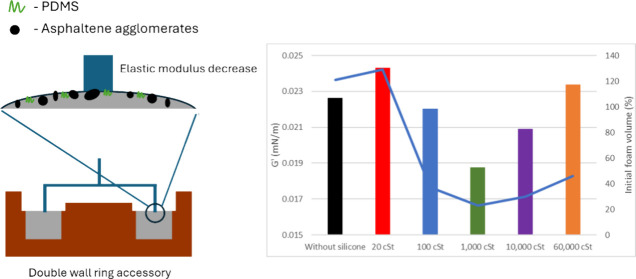

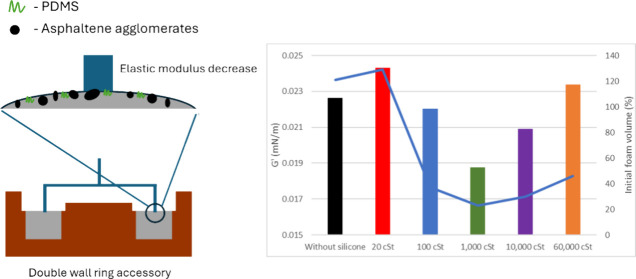

## 1 Introduction

The petroleum industry faces several challenges due to the existence of heavy fractions in its composition, including deposition of equipment, stabilization of foam and emulsion, and degradation of oil quality throughout the process. These issues not only hinder operational efficiency but also result in high maintenance costs, necessitating the incorporation of extra processes for optimal hydrocarbon recovery.^1–3^

Crude petroleum oil presents an intricate blend of hydrocarbons, encompassing light components like alkanes (linear, branched, and cyclic) and aromatics, alongside impurities such as sulfur, nitrogen, oxygen, and metals. Within the heavier fractions, noteworthy elements include asphaltenes, polar macromolecules that resist dissolution in saturated linear hydrocarbon of low molecular weight, and resins. The composition and ratios of these fractions fluctuate, impacting both the properties of the oil and the processes involved in refining it.^2,4,5^

The SARA method (Saturates, Aromatics, Resins, and Asphaltenes) categorizes petroleum fractions based on their solubility and polarity. Lighter fractions such as saturates (alkanes) and aromatics dissolve in aliphatic solvents, while heavier resins dissolve in both aliphatic and aromatic solvents. In contrast, despite having a structure similar to resins, asphaltenes, being the heaviest fraction with the most heteroatoms and metals and the highest molecular weight, remain insoluble in aliphatic solvents but soluble in aromatic solvents. These solubility distinctions play a fundamental role in petroleum refining, affecting deposit formation, viscosity, and product quality. The interplay between asphaltenes and resins, influenced by these solubility variations, becomes pivotal in optimizing production processes.^1,4–6^

Asphaltenes, solid compounds found in petroleum, tend to deposit in clusters due to their chemical structure.^7–10^ These asphaltenes exhibit amphiphilic behavior at the oil/air interface, imparting stability and elasticity to the interfacial film.^11–16^ Due to their amphiphilic molecular structure, asphaltenes exhibit a propensity for aggregate formation. This tendency arises from interactions among the polar moieties facilitated by van der Waals forces and hydrogen bonds, thereby fostering aggregation. The most potent intermolecular forces manifest as π-stack interactions between the abundant aromatic rings in the structure. The nonpolar segments, primarily composed of aliphatic chains, contribute to the stability of these aggregates. In conditions of elevated pressure and temperature, intermolecular forces play a fundamental role in promoting cohesion among asphaltenes, thereby enhancing their ability to form aggregates. These aggregates, in turn, give rise to the aforementioned consequences, such as the creation of solid deposits, blockage of conduits, and stabilization of emulsions.^1,4–6,17,18^

As asphaltene aggregates show intricate structures, two models address its stabilization. In the first model, aggregation occurs through resin adsorption on the asphaltene surface. Solvated resins induce a steric effect, restricting attractive van der Waals forces between asphaltene molecules and avoiding precipitation. The second model posits that asphaltenes’ interactions are predominantly influenced by medium solvation properties, with resins playing a negligible role.

Given their characteristics, asphaltenes exhibit specific intermolecular interactions in various solvents, displaying surfactant properties that stabilize water and oil emulsions. Also, asphaltene–resin complexes display adsorbance in the oil–water interface. Moreover, they contribute to the formation of a persistent oily foam, which is challenging to disrupt, leading to operational interruptions in extraction. Addressing this, antifoam agents like polydimethylsiloxane (PDMS) become essential.^2–4,6,18^

Currently, three main shear rheology techniques are being carried out to evaluate the behavior of the petroleum interfacial film in the literature. Among the techniques used is the Bicone accessory, used to study the mechanical properties of the asphaltene film formed at the oil/water interface^19^ and to evaluate the influence of surfactants at the oil/air interface.^20^ However, the Bicone accessory has high inertia, which is unsuitable for unstable films like the ones studied in this work. It results in low accuracy in data acquisition due to the drag of the mass phase.^21,22^

Another method used is the du Noüy ring, mostly applied in the study of water–oil emulsion dispersants, evaluating the rheological properties of interfacial films of model solutions and water with and without the presence of these additives.^23^ Bertelli and collaborators^24^ used this accessory to analyze the formation and subsequent inhibition of calcium naphthene at the water–oil interface. However, the du Noüy ring has certain limitations for shear measurements as the surface inside the ring is not considered and its round cross section is not ideal for fitting the fluid surface.^25^ Thus, the Double Wall Ring (DWR) can be considered an advancement for liquid shear and has a structure similar to the du Noüy ring. The du Noüy ring is composed of a 0.36 mm diameter Pt/Ir wire; however, the DWR has a diameter of 1 mm and sharp edges, providing an ideal shape for adjusting the accessory with the surface of the liquid. These changes allow for a better contact surface with the solution, increasing the sensitivity of the test and minimizing the effect of the mass phase on viscoelasticity.^26,27^ Therefore, DWR has been widely applied in the analysis of interfacial shear rheology.

Wang and collaborators^28^ evaluated the formation and breakdown of interfacial films with the DWR accessory for emulsions in the presence of polymers used in advanced oil recovery. There are also rheological assessment studies of the influence of asphaltenes on the oil/water interface.^14,29^ Also, Moradi and Alvarado^30^ analyzed the role of the ionic composition of water at the oil/water interface.

This study endeavors to assess the response of petroleum and its components (asphaltenes and maltenes) to PDMS products of varying viscosities in both crude oil and its fractions with a specific focus on the heavier asphaltene fractions using interfacial shear rheology. This technique enables examination of the mechanical response of interfaces between petroleum phases. Through the application of interfacial shear rheology, we aim to explore the interactions between asphaltene molecules and PDMS products across different shear conditions, temperatures, and compositions. This approach will provide insights into interfacial properties and elucidate the surfactant characteristics of petroleum components.^21,31^ By delving into these analyses, our objective is to comprehend how PDMS products, employed as antifoaming agents,^2^ engage with asphaltenes. The ultimate goal is to formulate effective strategies for mitigating the challenges encountered in oil production. This thorough understanding of the interactions and behaviors of petroleum components is vital not only for ensuring operational efficiency but also for maintaining the quality of petroleum production.

## 2 Experimental Section

### 2.1 Materials

Polydimethylsiloxane (PDMS) samples of different viscosities (20–60,000 cSt) were purchased from Sigma-Aldrich (Rio de Janeiro, RJ, Brazil). The characterization of the PDMS samples was carried out in a previous work.^32^ The solvents kerosene, toluene, and heptane were purchased from Isofar (Rio de Janeiro, RJ, Brazil). Two crude oil samples of different densities were donated by Centro de Pesquisas Leopoldo Américo Miguez de Mello (Cenpes) and Petrobras (Rio de Janeiro, RJ, Brazil). SARA characterization and viscosity of the oils were presented in a previous work.^32^ All samples were used as received.

### 2.2 Rheological Assessments for Viscosity

The viscosity values for crude oils 20 and 26° API and their derivatives were measured using a MARS 60 Haake rotational rheometer. The tests were conducted within a shear rate range (γ) of 50 to 400 s^–1^ at temperatures of 45 and 30 °C. The rheometer employed CC25 geometry (coaxial cylinders) coupled to a Huber thermostatic bath. In this apparatus, the rotor executes a rotational motion with a specified angular velocity, applying shear stress to the sample contained in the stationary vessel.

### 2.3 Extraction of Asphaltenes

Asphaltene samples from 20 and 26° API oils were extracted to generate model solutions to be used in rheological tests.

For extraction, approximately 30 g of oil was weighed, and 1 L of heptane was added. The mixture was stirred with a magnetic bar for 7 days.

After this period, the mixture was vacuum filtered three times by using the same filter. The maltenes were obtained by purifying the resulting liquid phase through rotary evaporation, which involved removal of the heptane.

The filtrate was placed in a cellulose cartridge and taken to the Sohxlet extractor to extract residual resins with the aid of 500 mL of heptane (Figure 1a).

**Figure 1:**
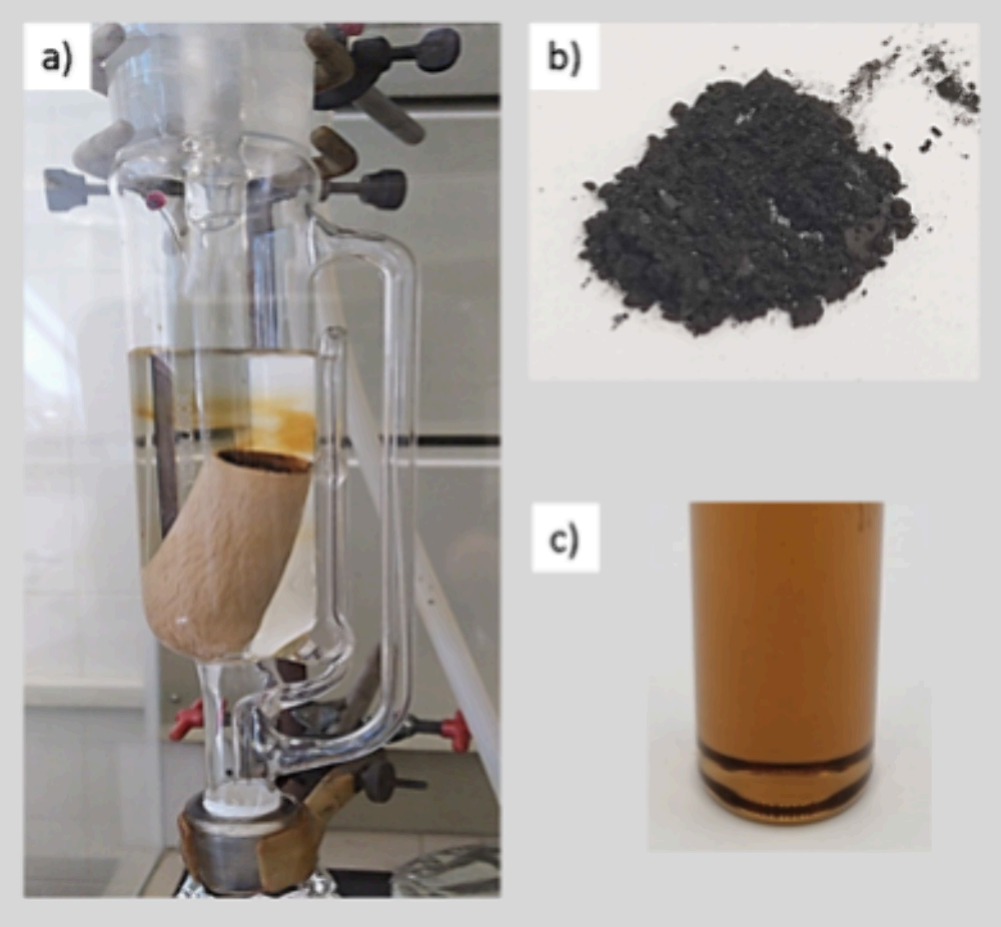
(a) Asphaltene extraction apparatus, (b) dried asphaltenes, and (c) model solution of asphaltenes: 0.05 wt % in Heptol 25/75.

Hereafter, the solvent was replaced by toluene, and the solubilization of the asphaltenes in the cartridge began. Subsequently, the toluene was evaporated, and the asphaltenes were stored in a dry place without the presence of light (Figure 1b).

The preparation of the model solutions was carried out with dispersions of asphaltenes with a concentration of 0.05% w/v in a mixture of solvents: 25% heptane/75% toluene, called Heptol 25/75 or model solution (Figure 1c).

### 2.4 Foam Formation Test

Foaming tests with crude oils and those containing antifoam products were presented in a previous work.^32^ In this review, we will also focus on foaming tests on maltenes. However, these tests containing asphaltenes in a model solution were not possible to carry out as there was no foam formation in the solution. It is believed that this is due to the low viscosity of the system, making foam formation impossible.

Foaming tests were performed by transferring 50 mL of maltenes into a 150 mL compression cell with or without the addition of an antifoam formulation. The antifoam formulations were prepared with 30 wt % of the active component polydimethylsiloxane (PDMS), dissolved in kerosene, and applied at a concentration of 20 ppm.

The cell underwent vigorous shaking for approximately 2 min (with or without the additive) followed by placement in a rotary oven for 120 min at temperatures of 45 and 30 °C for 20 and 26° API oil, respectively. Subsequently, the cell was pressurized with compressed air at 200 psi for 3 min. After pressurization, the cell was taken to the rolling oven for another 60 min.

The maltenes were then decompressed into a 100 mL beaker until a volume height of approximately 40 mL. A stopwatch was then started immediately to count the time for the formed foam to break, and the volume measurement was taken every 15 s until a constant value.

To calculate the foam content formed, eq 1 was used:

$$\mathrm{foam}\;(\%\mathrm{vol})=(V-V_{f})\times 100/V_{f}$$ 1

where *V* is the total volume reached by the oily foam on the scale at each time interval and *V*_f_ is the final height reached by the liquid after breaking all the foam formed.

### 2.5 Surface Rheology Studies

The main objective of this study was to observe the influence of formulations developed with antifoaming activity on the values of elastic and viscous modules of interfacial films (*G*′ and *G*″) since these properties are essential in understanding the mechanism of action of oil/air interfacial lamella.

Interfacial rheology analyses of oil samples, maltenes, and model solutions containing asphaltenes in Heptol 25/75 were carried out by the shear method using the TA Discovery Hybrid III rheometer. The rheological properties of the interfacial films formed were analyzed using the Double Wall Ring (DWR) accessory (Figure 2). The analysis chamber consists of a Teflon container with two walls of external radius 39.5 mm and internal radius 31 mm, which is placed on a Peltier plate. The ring, made of a Pt/Ir alloy, has a square cross section containing a sharp edge to form a planar interface.

**Figure 2:**
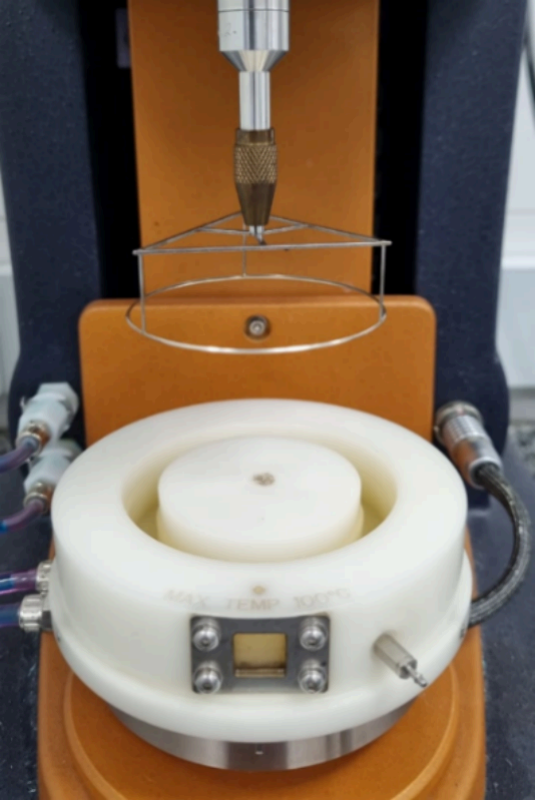
Double wall accessory and a Pt/Ir ring.

The temperatures of the tests with 20 and 26° API oils, as well as in the foaming tests, were 45 and 30 °C, respectively. And tests with maltene and asphaltene model solutions were carried out at 25 °C. All tests were carried out after aging for 2 h; the frequency was set at 15 Hz, the deformation was set at 10%, and the tests were performed as a function of time.

## 3 Results and Discussion

### 3.1 Rheological Assessments for Viscosity

Rheological testing was conducted on the oils within the shear rate range (γ) of 50 to 400 s^–1^ at 45 °C for 20 ° API and 30 °C for 26° API. The crude oils have a Newtonian behavior, as illustrated in Figure 3. The choice of temperatures was determined by the parameters employed in the antifoam test, in which a stable foam column was achieved for the surfactant application study. Within this region, the viscosity of the oil was unaffected by the selected shear rate.

**Figure 3:**
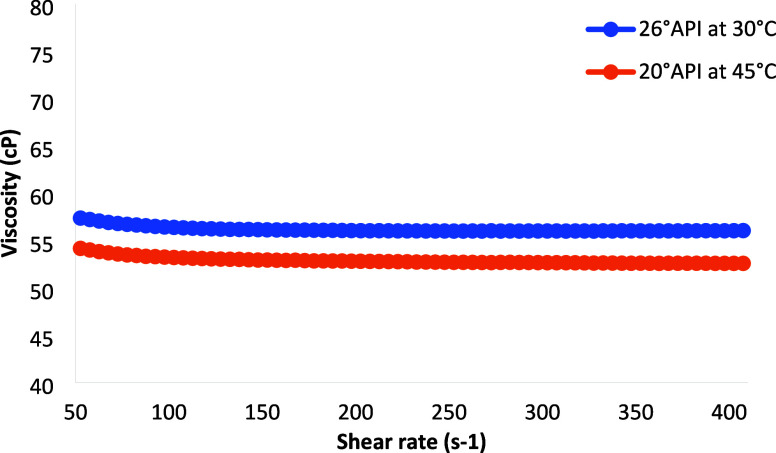
Viscosity curve of oils 26° API at 30 °C and 20 ° API at 45 °C.

The foam decay time is directly impacted by the viscosity of the oil, where a higher viscosity leads to a reduction in the drainage rate. Consequently, to facilitate a meaningful comparison of the behavior of both oils, temperatures were chosen to align their viscosities.^33^ Specifically, the 26° API oil displayed a viscosity of 57 cP at 30 °C, while the 20° API oil had a viscosity of 53 cP at 45 °C.

Viscosity assessments were conducted using maltenes derived from 20 and 26° API oils at the temperature of the foaming test. In both cases, they exhibited a Newtonian behavior.

### 3.2 Surface Rheology Studies

#### 3.2.1 Oil Samples

Given that the primary aim of this study is to assess the interfacial film of the systems using shear rheology with the DWR accessory, it is crucial to initially examine the strength of the interfacial film in oils without the addition of an antifoam formulation. The flow curves of all samples are shown in the Supporting Information. Figure 4 illustrates that the interfacial film of oil 20° API possesses an elastic modulus (*G*′) of about 0.019 mN/m, while in Figure 5, oil 26° API exhibits a *G*′ of about 0.023 mN/m.

**Figure 4:**
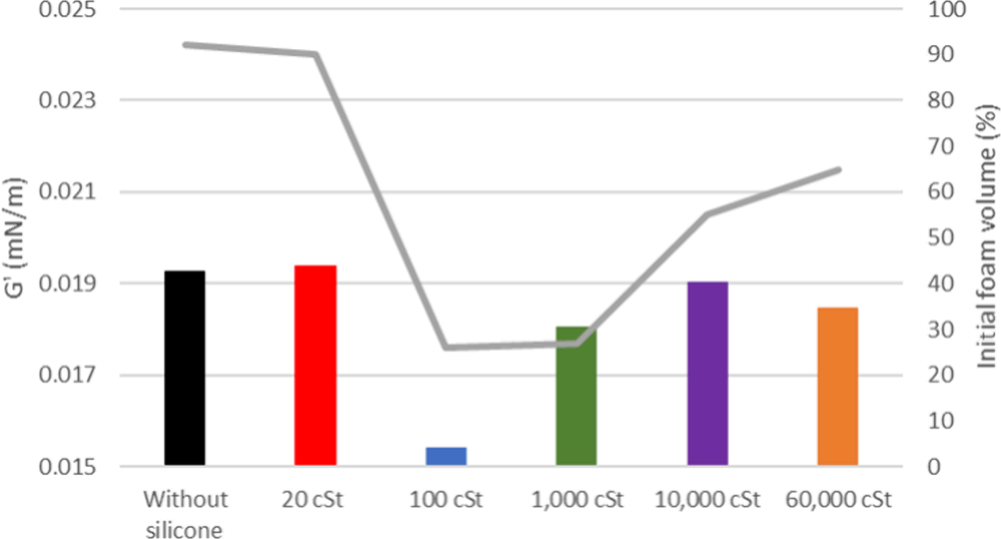
Elastic modulus of oil 20° API with and without silicone formulations (bars) and its respective initial formed foam (gray line).

**Figure 5:**
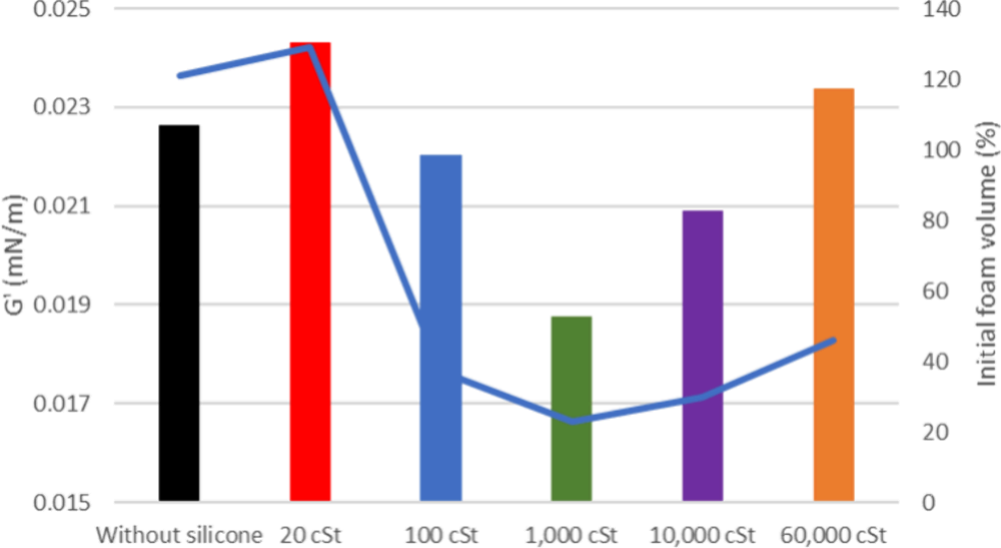
Elastic modulus of oil 26° API with and without silicone formulations and its respective initial formed foam (blue line).

The viscous modulus (*G*″) shows a slight increase for both 20 and 26° API oil when exposed to the samples, as depicted in Figures 6 and 7 (flow curves at the Supporting Information). It is noticeable that the *G*″ of the pure oil is smaller for the latest. The silicone samples show a similar change in *G*″ for 20° API oil except for 1000 cSt. As for 26° API, it shows a decreasing tendency for higher silicone molar masses.

**Figure 6:**
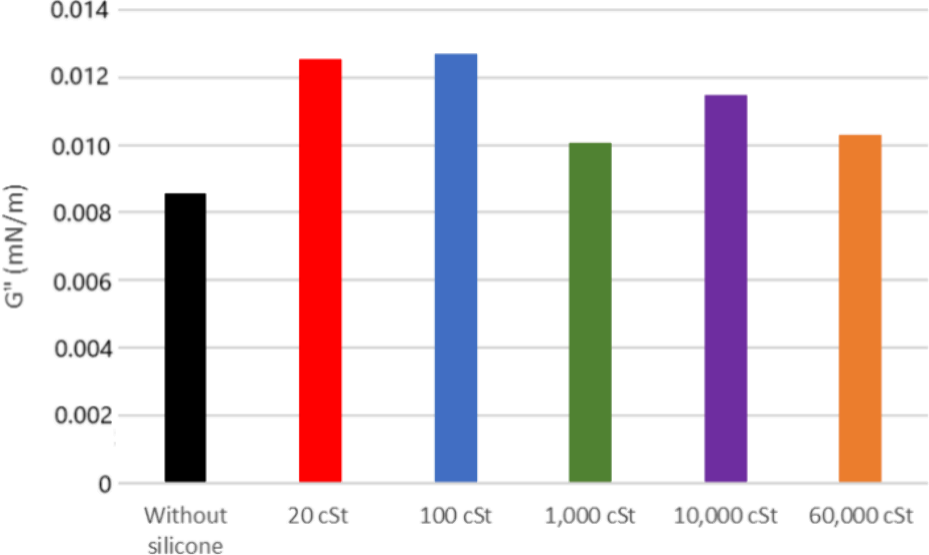
Viscous modulus of oil 20° API with and without silicone formulations.

**Figure 7:**
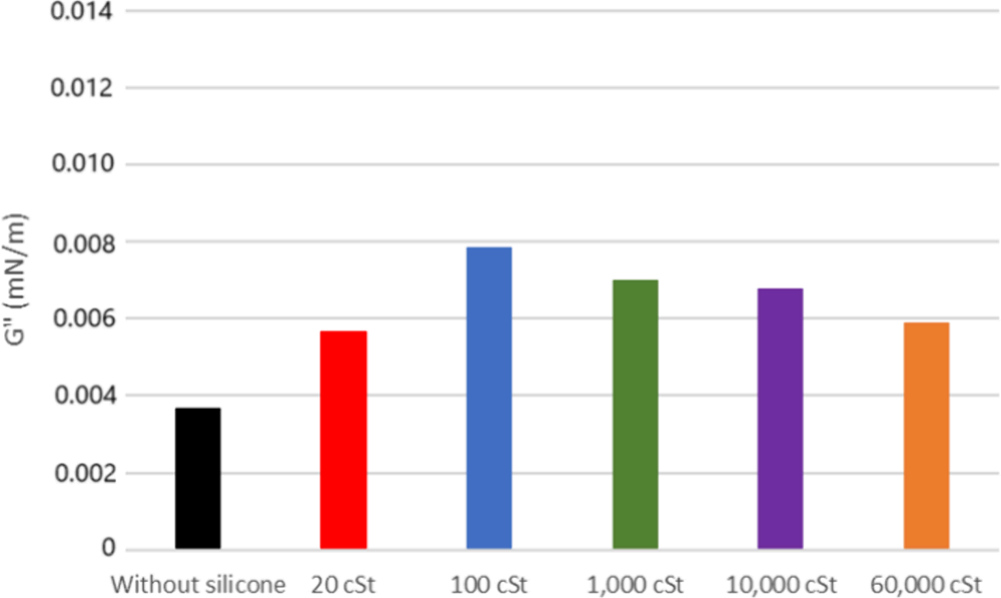
Viscous modulus of oil 26° API with and without silicone formulations.

The difference in composition between crude oils can be attributed to the different levels of asphaltenes and resins in these oils. The 26° API oil contains 2% asphaltenes and 20.3% resins, compared to the heavier oil (20° API) with 3.8% asphaltenes and 14.3% resins.^32^ The different interactions between resins and asphaltenes are believed to play a role in creating more substantial asphalt aggregates.^34–37^ Consequently, despite 26° API oil having a smaller amount of asphaltenes, the larger fraction of resins contributes to the development of more substantial and stable asphaltene clusters, which may migrate to the interface, resulting in a stronger interfacial film.

The line graphics depicted in Figures 4 and 5 illustrate the outcomes of foaming tests conducted on oil samples with API values of 20 and 26° API, respectively. The results are correlated with the initial foam height and are presented for both the presence and absence of silicone samples with varied viscosities (20, 100, 1000, 10,000, and 60,000 cSt).

The interfacial rheology results are consistent with the foaming test outcomes, which showed that the 26° API oil produced approximately 129% foam, while the 20° API oil generated about 92%. Although the experiments were conducted at different temperatures, both oils presented similar viscosities under the foaming test conditions. It is important to note, however, that these oils have distinct compositions, which may also influence foam formation. According to the literature, a reduction in foam stability is typically observed as viscosity decreases with increasing temperature,^38^ and the behavior of PDMS-based antifoams is known to vary depending on oil composition.^39^ Thus, the difference in the initial foam height is attributed not only to viscosity but also to the intrinsic foaming tendencies of the specific oil constituents under the experimental conditions.

When incorporating antifoam formulations, the alteration in the lamella’s robustness between the oil and air is observed. This highlights the direct impact of PDMS on the agglomeration of oil components that stabilize the interfacial film and their deposition in it. The inclusion of a formulation containing 20 cSt silicone led to an increased film strength (*G*′) at the interface in the case of 26° API oil, while no significant influence was observed for 20° API oil, aligning with the foaming test results. This is attributed to the molecular structure of the specific silicone, which is believed to be insufficiently large to hinder the formation of asphaltene clusters. The inclusion of PDMS 100 and 1000 cSt formulations resulted in a significant reduction of the interfacial film strength for 20 and 26° API oils, respectively. This pattern is similar to the foaming test results. It is hypothesized that this phenomenon arises from the viscosity disparity due to the molar mass. In the case of 26° API oil with higher fluidity (ρ = 0.8898 g mL^–1^), 1000 cSt silicone disperses more effectively, encountering less resistance in reaching the interface. This dispersibility is reinforced by the higher interference on *G*″ presented by this sample (Figure 7), possibly due to the higher interaction with asphaltenes in the bulk of the oil. On the other hand, for the more viscous 20° API oil (ρ = 0.9291 g mL^–1^), PDMS 100 cSt, with its smaller structure, exhibits smoother flow through the medium, reaching the interface more efficiently. This behavior hinders the agglomeration of asphaltenes in the interfacial film, consequently reducing its elasticity. *G*″ also presents higher values for this sample.

The effectiveness of the oil/air interfacial film strength in tests with 10,000 and 60,000 cSt silicones was not satisfactory as antifoam additives. These excessively large molecules lack the capacity to hinder the agglomeration of asphaltenes at the lamella interface. Consequently, this leads to heightened elasticity in the film, contributing to an increase in the value of *G*′. *G*″ present lower values for these samples as they may lack dispersibility in the bulk.

#### 3.2.2 Asphaltene and Maltene Samples

In pursuit of understanding the individual contributions of each petroleum component to the interfacial film’s elasticity, shear rheology tests were conducted separately for maltenes and asphaltenes. Figure 8 illustrates the model solution of 0.05% (m/v) asphaltenes in Heptol (25/75) from 20° API petroleum without the addition of PDMS, displaying an elastic modulus of 0.033 mN/m, the highest among the three systems studied (crude oil, model oil, and maltenes). The model solution of 0.05% (m/v) asphaltenes from 26° API, without the addition of PDMS, displayed an elastic modulus of 0.027 mN/m (Figure 10). This suggests that asphaltene agglomerates from heavier oil, likely influenced by their structure, have a tendency to be larger and more robust in comparison to those derived from lighter oil. This outcome supports the existing literature on asphaltenes, corroborating through shear rheology that they migrate and form clusters at the oil interface.^19,40,41^ By isolating the asphaltenes and keeping them at equivalent model solutions, eliminating other components that interact with the asphaltenes, and preventing their migration to the surface lamella, we can accurately evaluate the differences in the nature and resistance of asphaltene clusters from the different oils.

**Figure 8:**
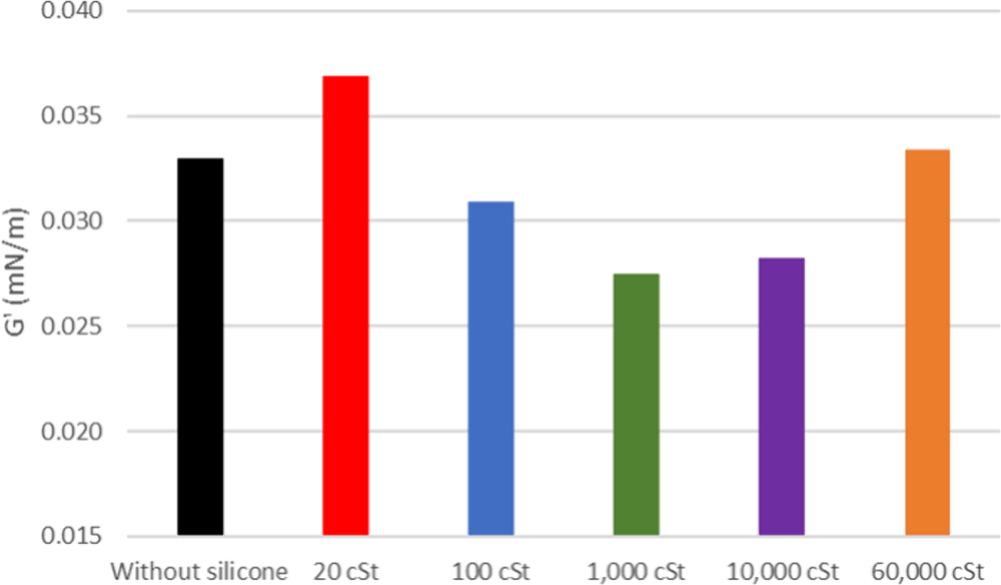
Elastic modulus of the model solution of asphaltenes from oil 20° API with and without silicone formulations.

**Figure 9:**
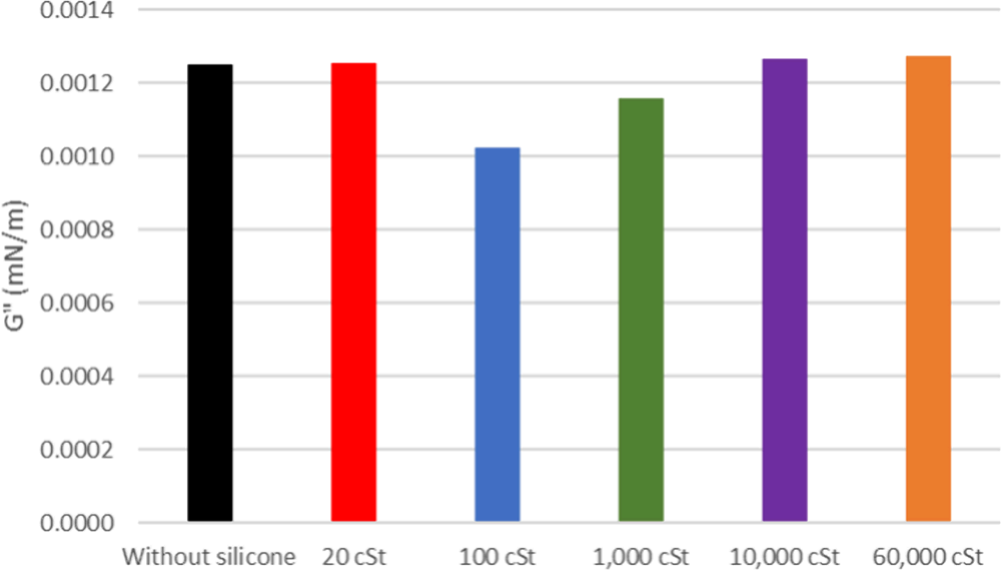
Viscous modulus of the model solution of asphaltenes from oil 20° API with and without silicone formulations.

As mentioned earlier, the product containing 100 cSt silicone demonstrated the most significant impact as an antifoam for 20° API oil, resulting in a reduction in the strength of the oil–air interfacial film. However, upon analyzing individual fractions, the formulation incorporating PDMS 1000 cSt exhibited a lower elastic modulus for the model solution of asphaltenes extracted from 20 and 26° API oils (*G*′ = 0.027 and 0.020 mN/m, respectively) (Figures 8 and 10). It is worth mentioning that the Heptol mixture without asphaltenes presented *G*′ = 0.027 mN/m, showing that this silicone oil was able to reduce the elasticity of the model solutions to the point where *G*′ is equivalent or inferior to the sample without asphaltenes. This decrease in *G*′ is likely attributed to the separation of the petroleum constituents—more specifically resins, a less viscous solution with fewer interferents—allowing the 1000 cSt silicone to disperse more effectively. This avoids the formation of asphaltene clusters in the model solution, leading to a reduction in the elasticity of the maltene interfacial film as the silicone migrates to the interface. The model solutions show no significant difference in *G*″ values (Figures 9 and 11).

**Figure 10:**
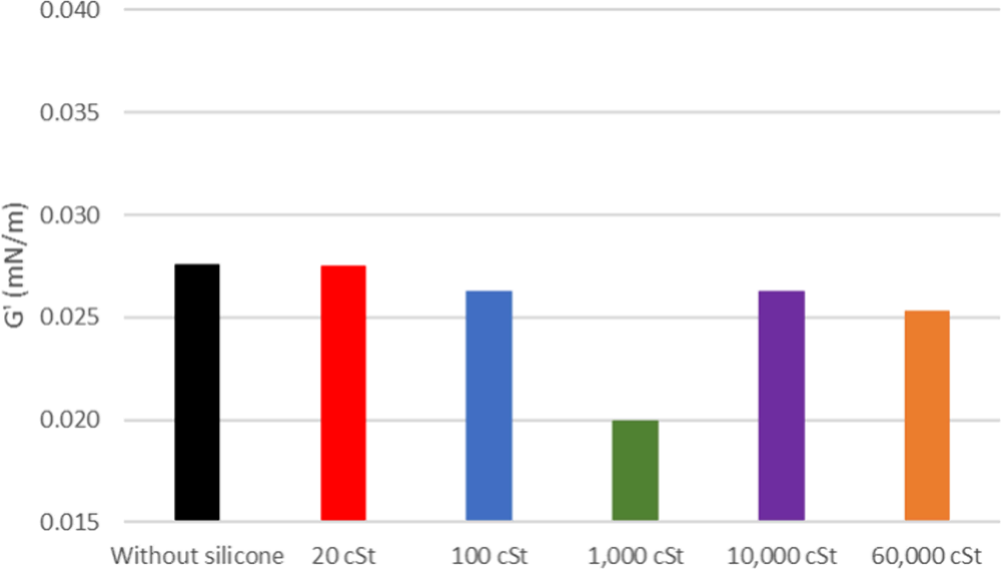
Elastic modulus of the model solution of asphaltenes from oil 26° API with and without silicone formulations.

**Figure 11:**
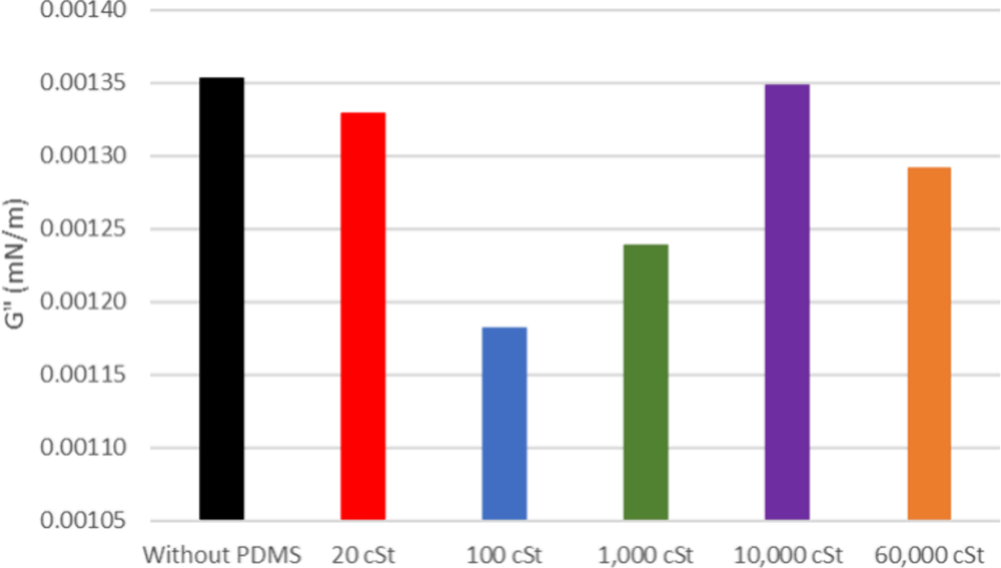
Viscous modulus of the model solution of asphaltenes from oil 26° API with and without silicone formulations.

The formulations with 10,000 and 60,000 cSt exhibited a similar behavior for the model solutions. However, there was an increase in the elastic modulus for the model solution added with the 20 cSt formulation compared with the pure model solution. In this context, the hypothesis posits that the mentioned silicone, given its lower molar mass compared with the studied PDMS variants, will exhibit a faster migration to the surface. This migration is expected to displace asphaltene molecules, leading to the product deposition at the interface and consequently enhancing the elasticity of the interfacial film.^42^ The Heptol mixture in the absence of asphaltenes was also tested in these conditions without the addition of PDMS (*G*′ = 0.0225 mN/m). When adding PDMS, independently of its viscosity, their interfacial behavior is alike (*G*′ ∼ 0.0275 mN/m), showing migration to the interface.

As depicted in Figure 12, the interfacial film strength of maltenes from 20° API petroleum, with and without additives, exhibits a higher value compared to its original oil. Despite C7 soluble asphaltenes being extracted, it shows higher interfacial stability most likely from the remaining resins. Once again, the 1000 cSt sample has presented lower interfacial activity followed by 100 cSt. The viscous modulus (Figure 13) shows an increase when silicone is added but no significant difference between results.

**Figure 12:**
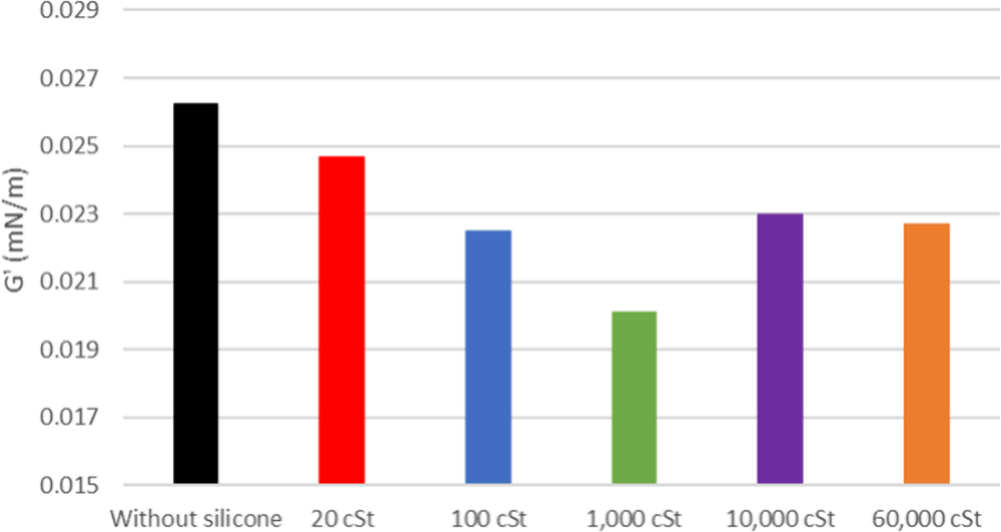
Elastic modulus of maltenes from oil 20° API with and without silicone formulations.

**Figure 13:**
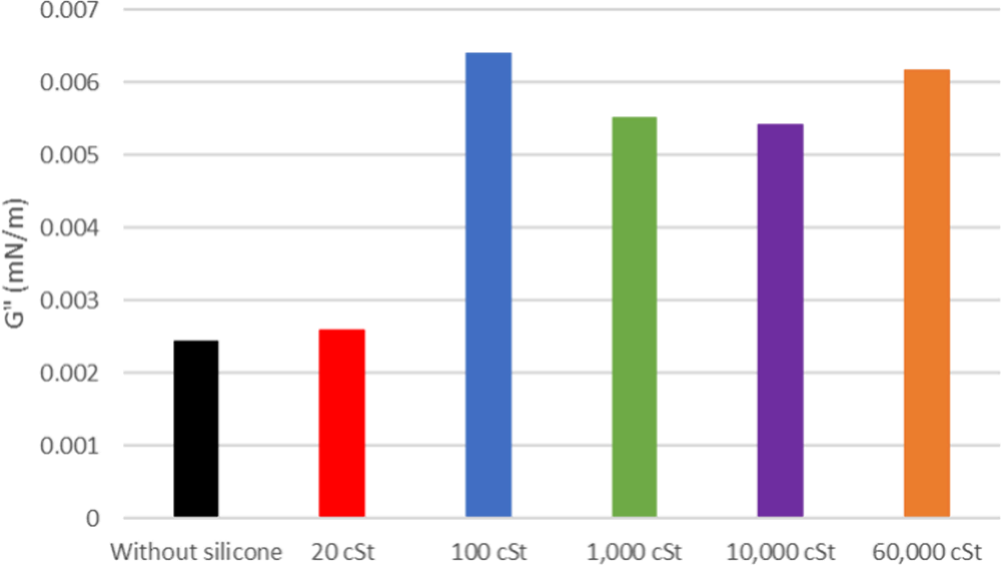
Viscous modulus of maltenes from oil 20° API with and without silicone formulations.

For maltenes from 26° API oil, the profile is more similar to the oil of origin, with 1000 cSt silicone at lower *G*′, as presented in Figure 14, configuring the same behavior as 20° API oil. The viscous modulus also shows a minor increase when silicone is added, except for the 10,000 cSt sample, seemingly a deviation from the pattern (Figure 15).

**Figure 14:**
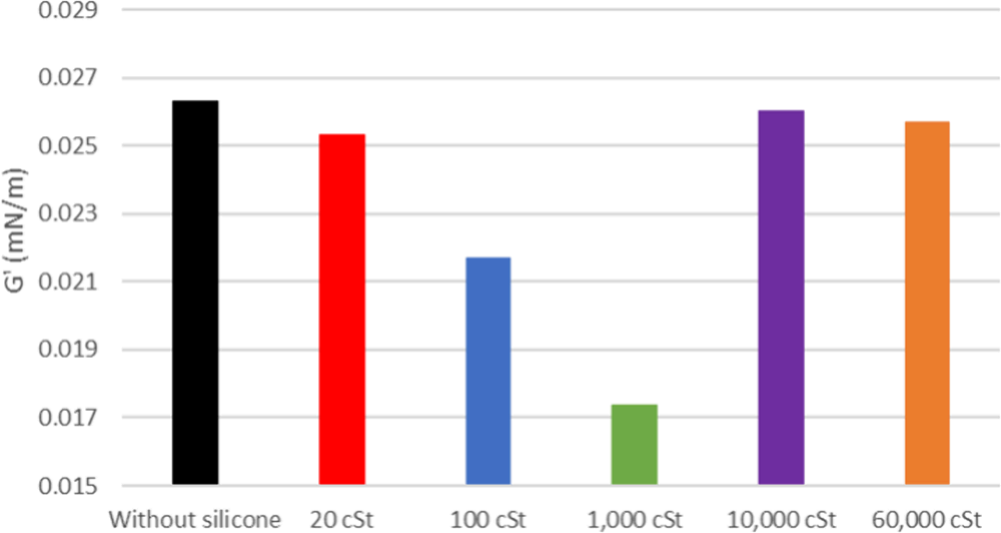
Elastic modulus of maltenes from oil at 26° API with and without silicone formulations.

**Figure 15:**
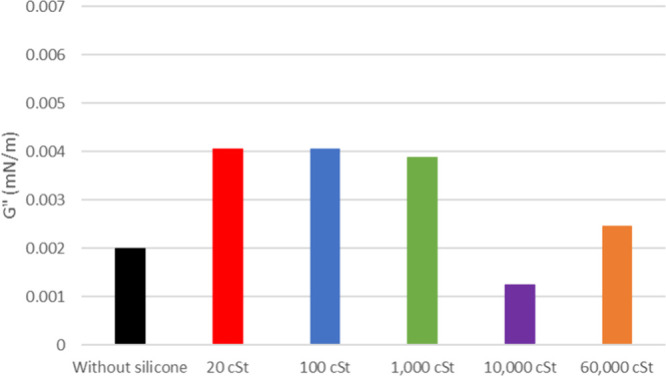
Viscous modulus of maltenes from oil 26° API with and without silicone formulations.

Remarkably, the foaminess of maltenes from 20 and 26° API oils without additives, assumably originated from resins, was noted once asphaltenes were extracted. As elastic moduli were higher than those observed for the oils, the resins, also natural foamers in petroleum, should generate even more stability for the oil film than the asphaltenes.

There was no noteworthy distinction observed among maltene samples concerning the initial foam volume formed. The antifoam effect from silicone oil was tested on maltenes, specifically the 100 cSt sample, showing a decrease of about 90% for 20° API and 82% for 26° API (Supporting Information Figures S13 and S14).

Finally, the observation of silicone interaction with fractions of the oil through elastic moduli shows a similar behavior to those tests with silicone in oil in natura. For 20° API, the 1000 cSt silicones show lower *G*′ values for the model solution and maltenes, whereas for the oil, the 100 cSt sample has smaller *G*′, indicating more interfacial activity on weakening the oil film stability. This may occur to a 100 cSt sample because the interactions between asphaltenes and their natural dispersants, the resins, interfere with silicone access to these asphaltenes.

For 26° API, the 1000 cSt silicone shows lower *G*′ for all three cases, indicating that this range of molar mass (100 to 1000 cSt) shows higher access to the interface independently of how much asphaltenes are present in the oil composition. As *G*″ is virtually equal for the analyzed cases, the added silicone does not affect viscosity.

## 4 Conclusions

The utilization of shear rheology tests with the DWR accessory has demonstrated effectiveness in the selection of a defoamer for petroleum products. The tests conducted in this study revealed that the product causing the most significant reduction in the elastic modulus also exhibited the lowest initial foam height.

The presented scenario shows the importance of the composition of the oil, specifically the resin/asphaltenic ratio, on the choice of which PDMS is better suited as an antifoamer agent. It is evident through rheological analysis of the surface of the three systems that asphaltenes tend to aggregate on the surface, be it in oil or in the model solution. Also, resins present in maltenes have a role in foam stabilization.

## Supplementary Material



## Notes

Flow curves for oil 20 and 26° API for the model solution of asphaltenes from 20 and 26° API oils and maltenes extracted from 20 and 26° API oils and graphics of formed foam vs time for maltene samples (PDF)
