# Supplementary material for: Assessment of Asphaltene and Maltene Interfacial Interactions with Antifoam Additives: A Rheological Approach
Source: ACS Omega. 2025 May 14;10(20):20515–23. doi: 10.1021/acsomega.5c00847 (PMC12120640; doi:10.1021/acsomega.5c00847)
Supplement: Supplementary file 1 [file ao5c00847_si_001.pdf]

Supplementary Material

ASSESSMENT OF ASPHALTENE AND MALTENES INTERFACIAL INTERACTIONS WITH ANTIFOAM ADDITIVES: A RHEOLOGICAL APPROACH

Mariana T. Mendes[1], Andressa O. dos Santos[1], Rafael F. Perez[1*], Osvaldo Karnitz Junior[3] and Claudia R. E. Mansur[1,2]

[1] Universidade Federal do Rio de Janeiro, Programa de Engenharia Metalúrgica e de Materiais-PEMM/COPPE. Av. Horácio Macedo, 2030 - Bloco F-CT, Cidade Universitária, Rio de Janeiro – RJ, 21941-598, Brazil.

[2] Universidade Federal do Rio de Janeiro, Instituto de Macromoléculas/Laboratório de Macromoléculas e Colóides na Indústria de Petróleo. Rua Moniz Aragão, 360. Bloco 8G-CT2, Cidade Universitária, Rio de Janeiro – RJ, 21941-594, Brazil.

[3] Leopoldo Américo Miguez de Mello Research and Development Center
Rio de Janeiro, RJ, BR 21941-598, Brazil.

*rafaelfperez@ima.ufrj.br

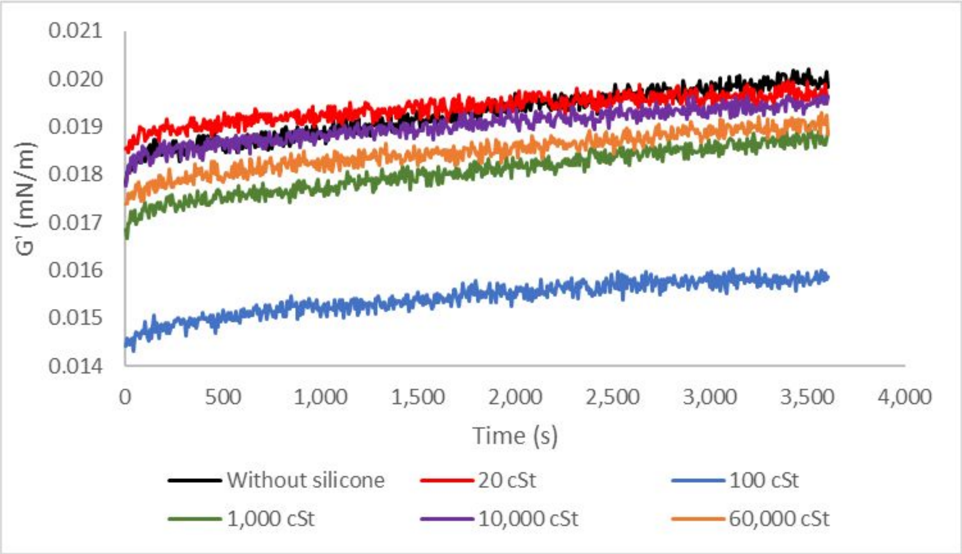

Figure S1. Elastic modulus (G') of oil 20 °API pure and with antifoam formulations.

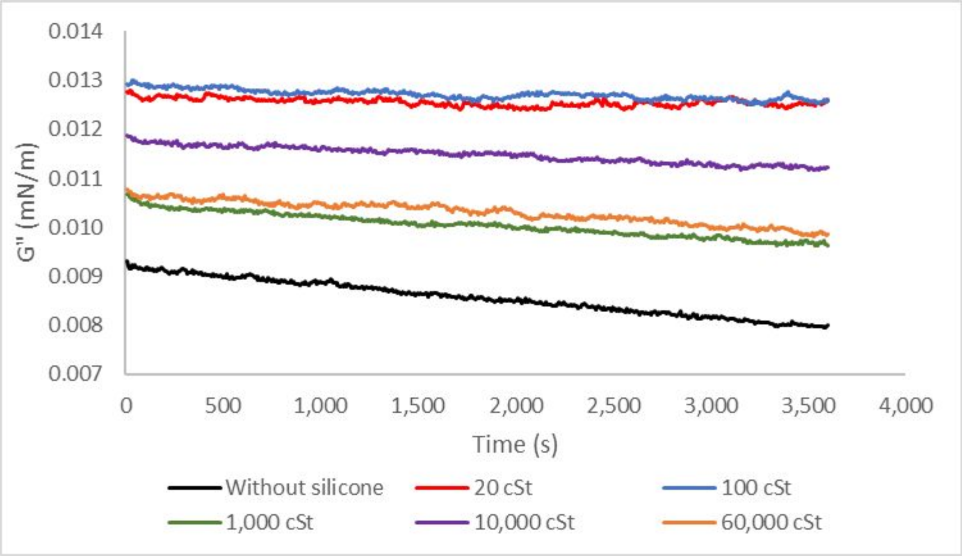

Figure S2. Viscous modulus (G”) of oil 20 °API pure and with antifoam formulations.

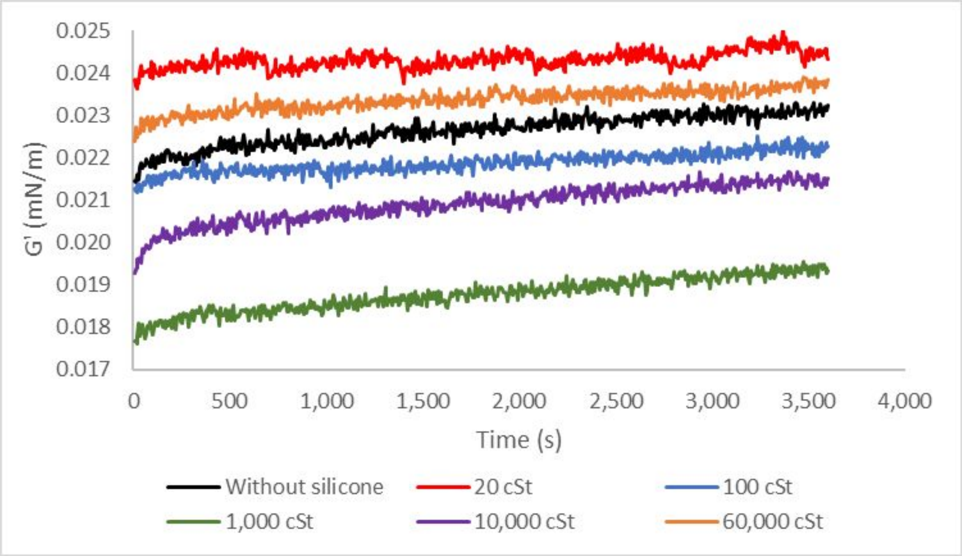

Figure S3. Elastic modulus (G’) of oil 26 °API pure and with antifoam formulations.

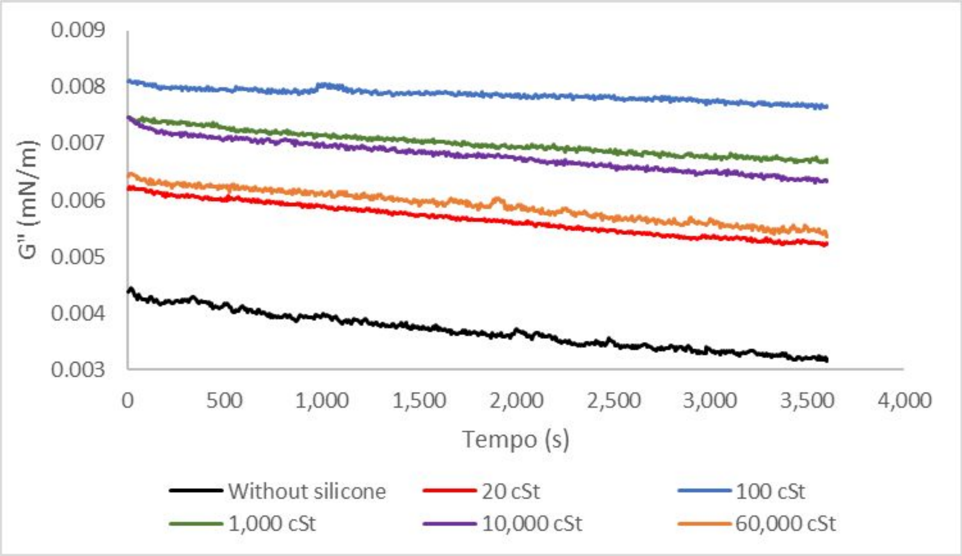

Figure S4. Viscous modulus (G”) of oil 26 °API pure and with antifoam formulations.

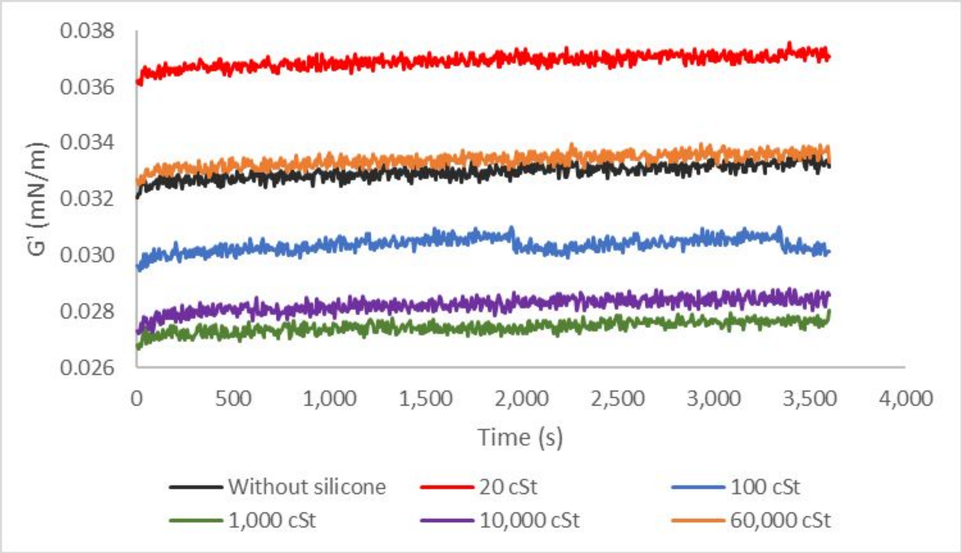

Figure S5. Elastic modulus (G’) of model solution of asphaltenes from 20 °API pure, and with antifoam formulations.

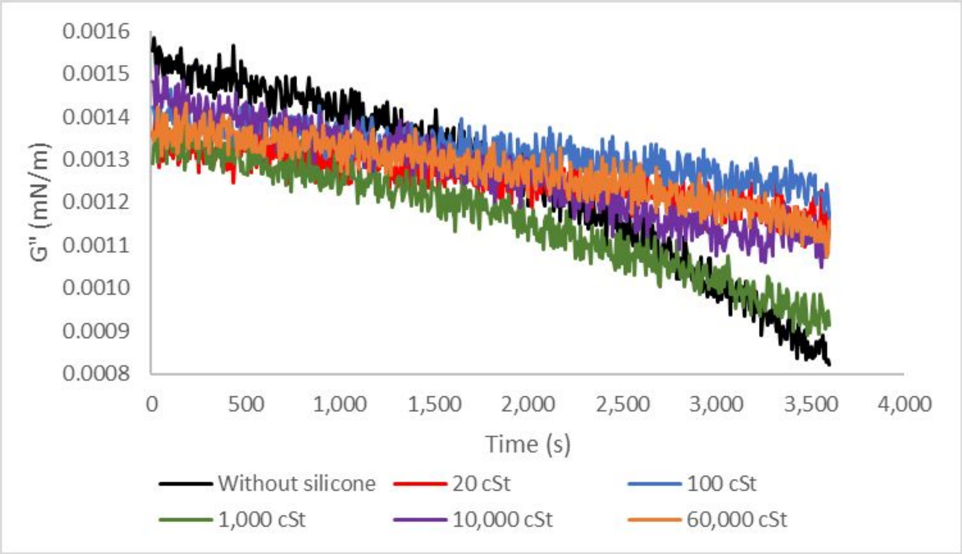

Figure S6. Viscous modulus (G”) of model solution of asphaltenes from 20 °API pure, and with antifoam formulations.

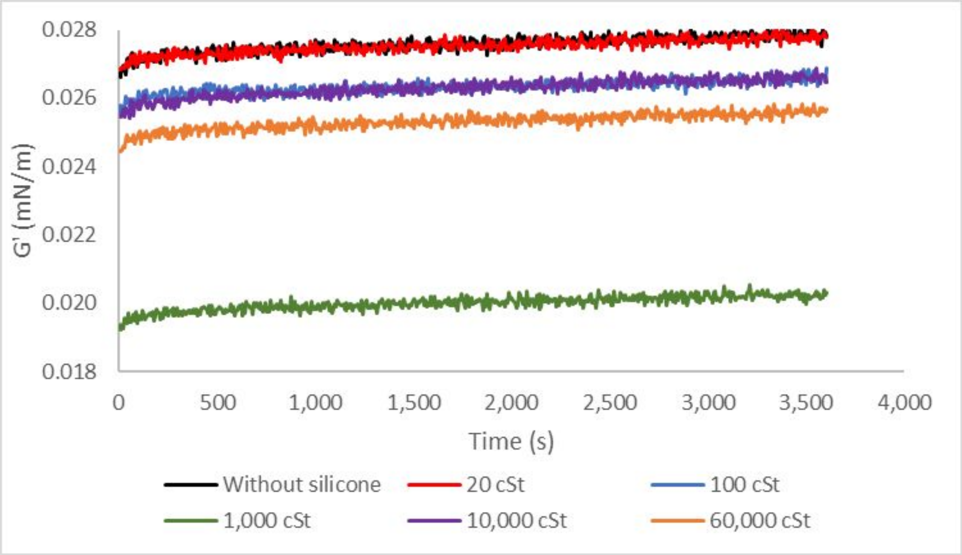

Figure S7. Elastic modulus (G’) of model solution of asphaltenes from 26 °API pure, and with antifoam formulations.

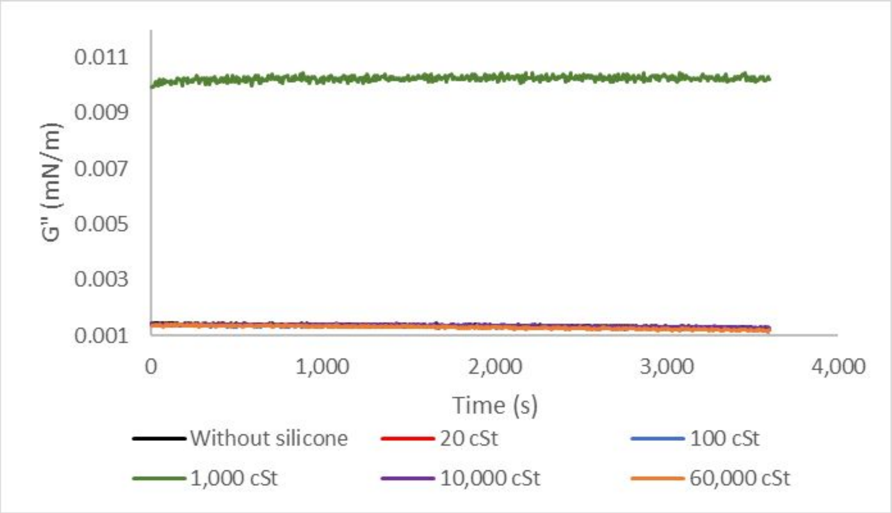

Figure S8. Viscous modulus (G”) of model solution of asphaltenes from 26 °API pure, and with antifoam formulations.

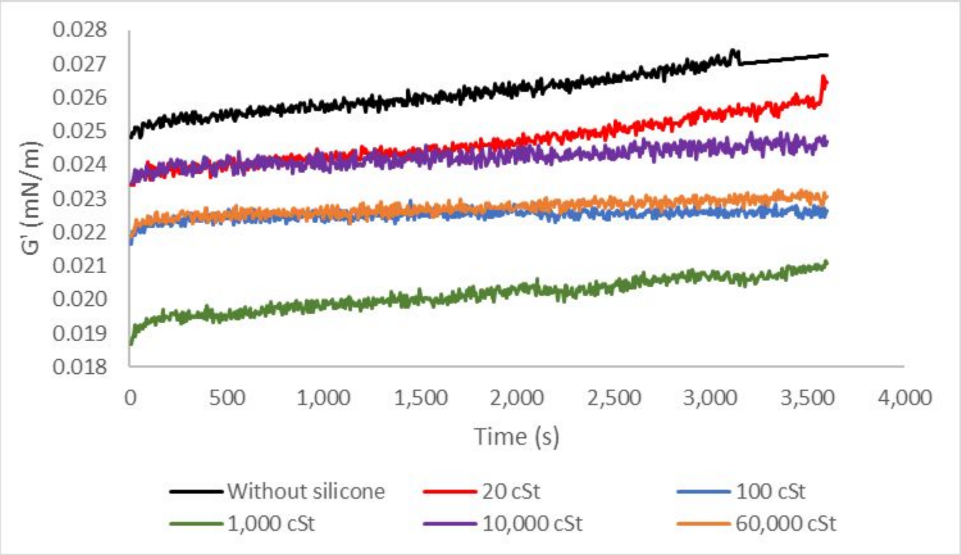

Figure S9. Elastic modulus (G’) of maltenes extracted from 20 °API, pure and with antifoam formulations.

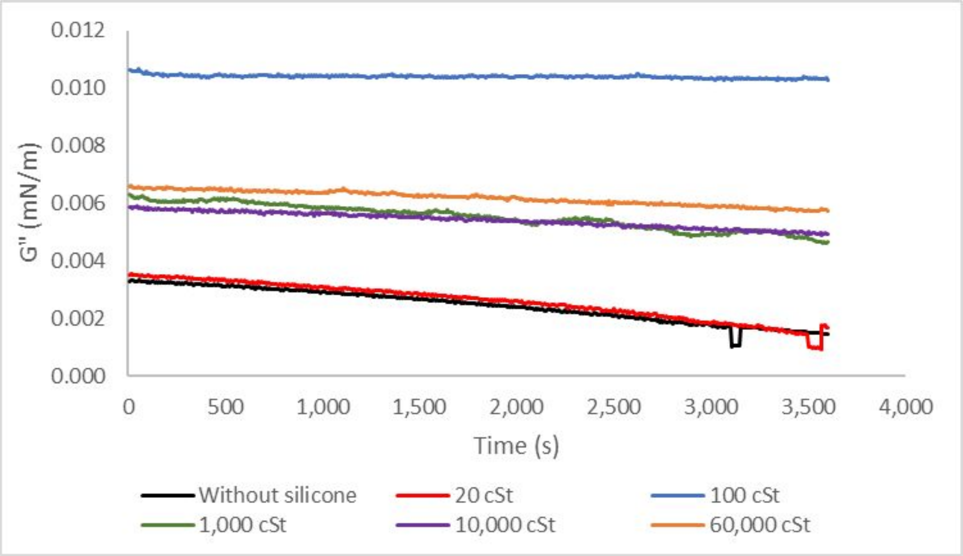

Figure S10. Viscous modulus (G”) of maltenes extracted from 20 °API, pure and with antifoam formulations.

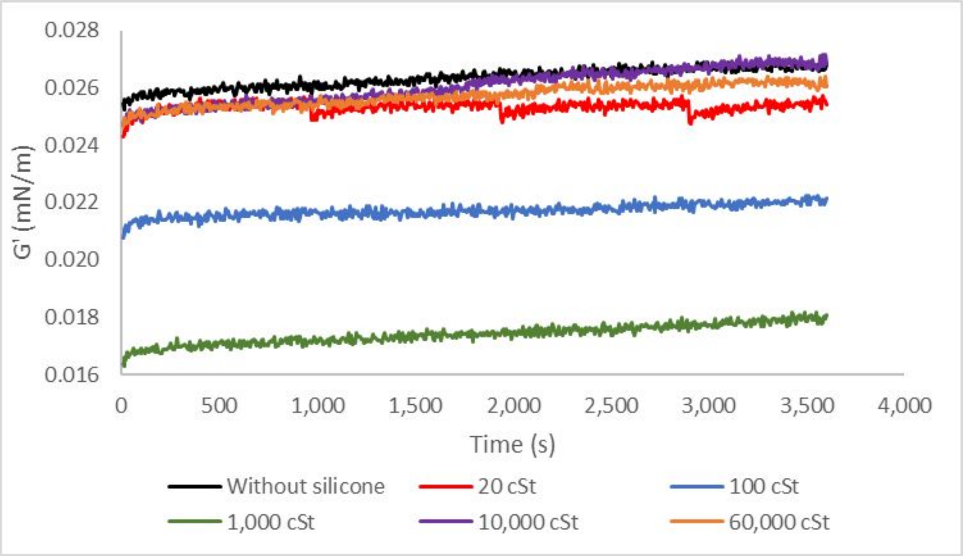

Figure S11. Elastic modulus (G’) of maltenes extracted from 26 °API, pure and with antifoam formulations.

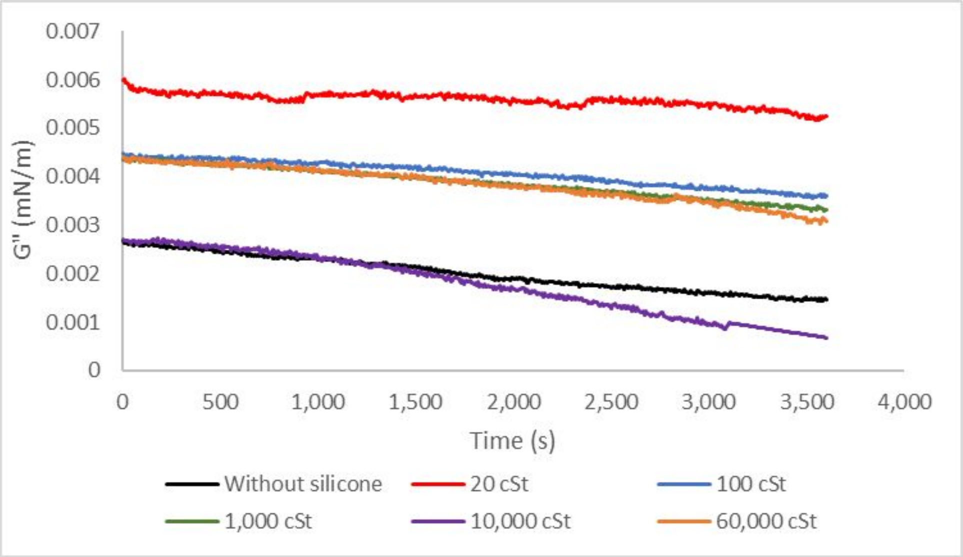

Figure S12. Viscous modulus (G”) of maltenes extracted from 26 °API, pure and with antifoam formulations.

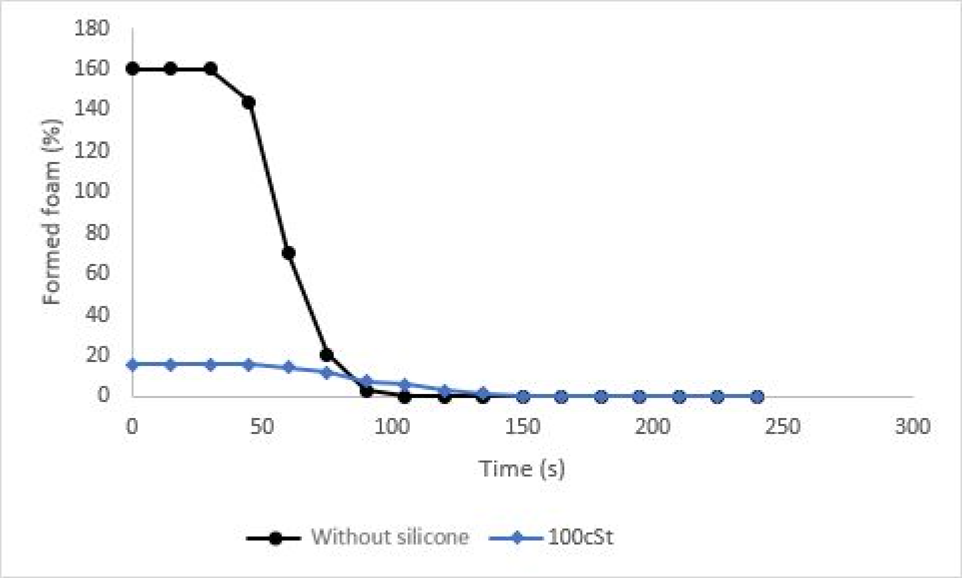

Figure S13. Formed foam over time of maltenes extracted from 20 °API, pure and with antifoam formulations.

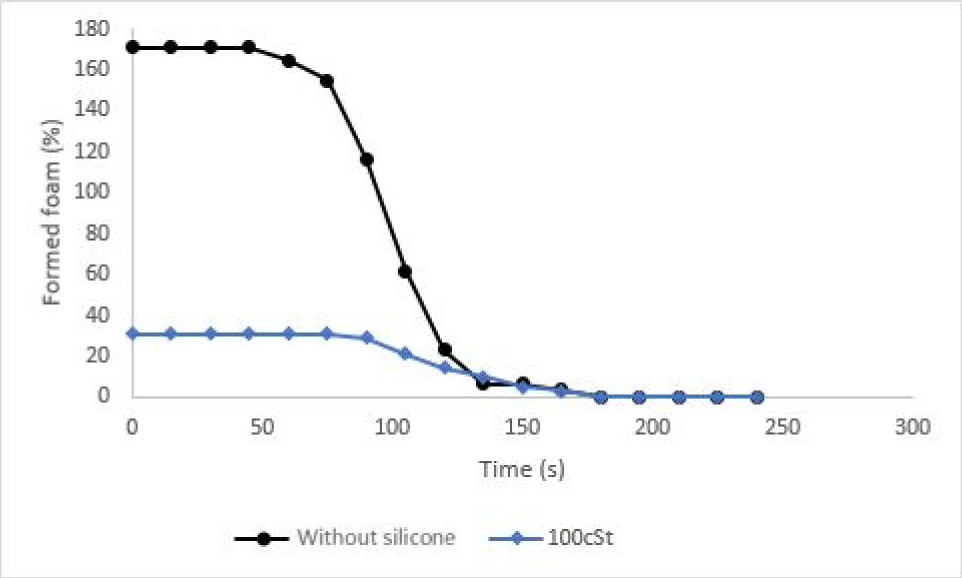

Figure S14. Formed foam over time of maltenes extracted from 26 °API, pure and with antifoam formulations.
